# RNF213 marks a regulatory T-cell subset associated with ulcerative colitis

**DOI:** 10.3389/fimmu.2026.1871605

**Published:** 2026-07-06

**Authors:** Zhangqin Li, Jing Wu, Jiayi Yang, Lianyu Zhou, Lifang Chen, Youxu Ren, Yunxing Li, Ruijie Ma, Yinglei Miao, Jie Jia, Jiarong Miao

**Affiliations:** 1 Department of Gastroenterology, First Affiliated Hospital of Kunming Medical University, Kunming, China; 2 Yunnan Province Clinical Research Center for Digestive Diseases, First Affiliated Hospital of Kunming Medical University, Kunming, China; 3 Department of Gastroenterology, The People’s Hospital of Xishuangbanna Dai Nationality Autonomous Prefecture, Xishuangbanna, China; 4 Research Center for Clinical Medicine, First Affiliated Hospital of Kunming Medical University, Kunming, China

**Keywords:** inflammation, RNF213, regulatory T cells, ubiquitination, ulcerative colitis

## Abstract

**Background:**

Ulcerative colitis (UC) is a nonspecific autoimmune disease, the etiology and pathogenesis of which remain unclear. Identifying new disease targets could inform clinical diagnosis and treatment.

**Methods:**

We integrated single-cell RNA sequencing (scRNA-seq) and bulk transcriptome data from UC patients. We screened key biomarkers and constructed a nomogram diagnostic model through three machine learning algorithms (LASSO, SVM-RFE, and Boruta). *In situ* validation was conducted on mucosal tissue sections from clinical UC and healthy controls using multiplex immunofluorescence (mIF). To investigate the intracellular mechanisms by which RNF213 regulates Tregs, we employed scTenifoldKnk and CellChat analyses. Potential targeted compounds were predicted based on the DSigDB and HERB databases.

**Results:**

Five UC-specific ubiquitination biomarkers were identified: ZC3H12A, ENC1, RNF213, MAP3K5, and RMND5A. Among them, RNF213 demonstrated superior diagnostic performance with an AUC greater than 0.9 in both training and validation cohorts. Single-cell localization and *in situ* mIF quantification indicated that RNF213 was enriched in mucosal Tregs from UC patients. *RNF213^+^* Tregs were increased in UC compared with healthy controls. *In-silico* RNF213 perturbation analysis suggested that RNF213-enriched Tregs may be associated with transcriptional programs related to mitochondrial proton transport and ATP synthesis. CellChat analysis further predicted altered ligand-receptor interaction patterns among *RNF213^+^* Tregs and other immune cell populations, including monocytes, macrophages, and B cells.

**Conclusions:**

This study identifies RNF213 as a biomarker enriched in UC-associated mucosal Tregs. *RNF213^+^* Tregs may represent a transcriptionally and spatially distinct Treg subset associated with inflammatory activity in UC.

## 1 Introduction

UC is a chronic nonspecific inflammation of the intestinal tract. With the Westernization of diet, rapid urbanization, and significant change in environment, UC incidence has increased steadily (1, 2). Global hospitalization trends for Crohn’s disease and ulcerative colitis have also changed substantially in the 21st century (3). The disease often continues its course and is more likely to lead to colorectal cancer (4). In the biologics era, the treatments have grown over the last decade to include anti-tumor necrosis factor drugs (TNF), vedolizumab, ustekinumab, and small molecules (Janus kinase inhibitors and sphingosine-1 phosphate receptor modulators (2), but still face challenges like low efficacy and negative consequences such as infections (5). Elucidating the pathogenic mechanisms of UC is essential for informing clinical management and guiding the development of targeted therapies.

The UC pathogenesis is still poorly understood, and immune dysregulation is central to disease development (2). Balance among T-cell subsets such as Th17 and regulatory T cells (Tregs) is critical for intestinal immune homeostasis in UC. Tregs are *CD4*^+^*CD25*^+^*FOXP3*^+^ expressing IL-10 and TGF-β and contact-dependently suppress excess immunity. Multiple studies report increased Th17 numbers in colonic mucosa of UC patients with decreased Treg frequency or impaired Treg function (6). When severely inflamed, Tregs may turn into “inflammatory Tregs” producing proinflammatory mediators (*IL-8*^+^*Foxp3*^+^ T cells), which worsen tissue damage (7). Functional plasticity is controlled by several molecular switches, among which ubiquitin-mediated degradation of Foxp3 is one of the major triggers of Treg inactivation. scRNA-seq identifies exact populations of intestinal cells (fibroblasts, macrophages, epithelium, and T cells) with high resolution (8, 9). We integrated scRNA-seq and transcriptomic data from UC samples, applied two-dimensional dimensionality reduction to Treg cells, and combined machine learning to conduct diagnostic validation of biological targets. Through this approach, we identified potential diagnostics for UC.

Ubiquitination, the key control over degradation and signal transmission of proteins, has recently been studied in the study of inflammatory bowel disease. Balance of ubiquitination and deubiquitination by DEBUs is important for maintaining colonic function in UC. In inflammatory bowel disease, target protein function depends on the linkage of chain linkage: K63-linked chains (such as those produced by RNF213) promote FOXO1 nuclear translocation and thus Treg differentiation (10), and linear ubiquitin chains activate the NF-ĸB pathway and are closely associated with NLRP3 activation (11).

We integrated multiple scRNA-seq and bulk transcriptomic datasets from the Gene Expression Omnibus (GEO) and applied machine learning algorithms to identify RNF213 as a key gene associated with UC. RNF213 is currently the only known molecule with AAA^+^ ATPase and E3 ubiquitin ligase activity (12). RNF213 mediates K63-linked ubiquitination of the FOXO1 promoter that induces FOXO1 nuclear translocation. Nuclear FOXO1 binds and activates the Foxp3 promoter, leading to Treg differentiation in moyamoya disease. However, to date, the relationship between UC and RNF213 has never been reported.

Through the integrative analysis of multiple UC datasets, RNF213 was identified as a ubiquitination-related candidate gene enriched in UC-associated Tregs, suggesting its potential relevance to Treg remodeling and biomarker discovery in UC.

## 2 Materials and methods

### 2.1 Clinical information

All patients had undergone 14 weeks of induction therapy with infliximab. We used the modified Mayo score and the Mayo Endoscopic Subscore (MES) to distinguish disease activity and collected patient clinical information to comprehensively assess disease characteristics, and the study protocol was approved by the Ethics Committee of the First Affiliated Hospital of Kunming Medical University (ethics approval number: 2024L274).

### 2.2 Data resource

The transcriptomic data were downloaded from GEO (http://www.ncbi.nlm.nih.gov/geo/). The GSE87466 dataset (87 UC and 21 control samples) was used as training data, and the GSE75214 (97 UC and 11 control samples) was used for validation. The scRNA-seq data (GSE214695 and GSE182270) were to resolve heterogeneity. The list of 1,363 UbRGs was obtained from iUUCD 2.0 (http://iuucd.biocuckoo.org/).

### 2.3 Data analysis

The Seurat package is used for processing scRNA-seq data. Cell clusters are tagged with the Cell Marker database and lineage-specific markers. Differential expression genes (DEGs) analysis is performed on bulk RNA-seq data using the “limma” package with *log2|FC| >0.5* and *P-value < 0.05*. Three machine learning algorithms are used to find diagnostic biomarkers: LASSO regression, SVM-RFE and the Boruta algorithm. Using the R package scTenifoldKnk to knock out RNF213 in Treg cells, followed by functional enrichment analysis with the R package clusterProfiler.

### 2.4 Multiplex immunofluorescence and imaging analysis

To validate spatial expression of key biomarker RNF213. mIF was performed on colonic mucosa sections of UC patients (moderate to severe UC: *n* = 4 and mild UC: *n* = 4) and healthy controls (*n* = 4). For each sample, they were quantified using HALO software under identical threshold settings across all samples. DAPI-positive nuclei were used to define total cells. Tregs were identified as CD4^+^*CD25^+^ FOXP3^+^* cells, and *RNF213^+^* Tregs were identified as *CD4^+^ CD25^+^ FOXP3^+^* cells with positive RNF213 staining. The frequencies of *RNF213^+^* Tregs among *CD4^+^* T cells and total Tregs were calculated as *RNF213^+^ CD4^+^ CD25^+^ FOXP3^+^* cells/total *CD4^+^ CD25^+^* cells × 100%. And *RNF213^+^ CD4^+^ CD25^+^ FOXP3^+^* cells/total *CD4^+^ CD25^+^ FOXP3^+^* cells × 100%, respectively. All quantification was independently performed by two investigators blinded to clinical grouping. The antibodies used were CD4 (Abcam, Catalog No. ab288742), CD25 (Abcam, Catalog No. ab231441), FOXP3 (Proteintech, Catalog No. 22228), and RNF213 (Invitrogen, Catalog No. PA5-145785).

### 2.5 Statistical analysis

The statistical analyses were performed in R (v4.2.2). Comparisons between two groups were performed using the Wilcoxon rank-sum test. Quantitative mIF results were analyzed using GraphPad Prism 10.0. Statistical homogeneity of variance between the three groups was tested by one-way ANOVA, in which homoscedasticity was satisfied. *ns*, *P* > 0.05; *P < 0.05; **P < 0.01; ***P < 0.001; ****P < 0.0001.

## 3 Results

### 3.1 Clinical characteristics of the participants

**Figure 1:**
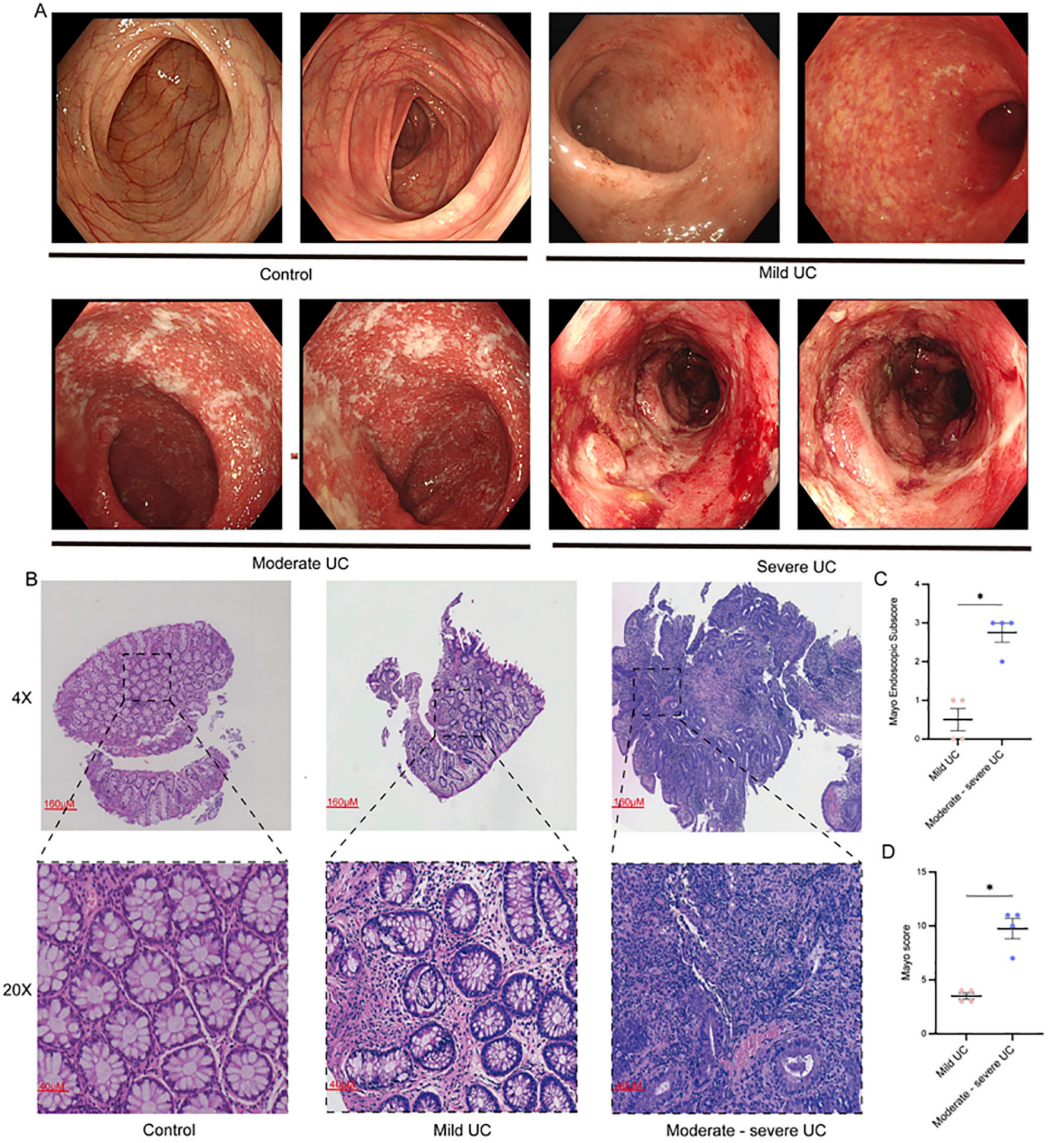
**(A)** Typical endoscopic images from colonoscopy of healthy individuals and UC patients; **(B)** Histopathological findings in UC patients; **(C)** Statistical graph of MES; **(D)** Statistical graph of Mayo score. ^*^P < 0.05; ^***^P < 0.001.

The healthy control group and patients with UC were matched for baseline characteristics of age and sex, with no statistically significant differences. Mucosal samples from UC (*n* = 8) and healthy control (*n* = 4) were collected for H&E staining. The mean disease duration was 5.25 ± 1.377 years in the moderate to severe UC and 6.25 ± 0.75 years in the mild UC (*P* > 0.05). The disease extent was pancolitis in all patients. Clinical parameters, including erythrocyte sedimentation rate (ESR), platelet count (PLT, × 10^9^/L), and white blood cell count (WBC, × 10^9^/L), were consistent with disease activity indices (MES and Mayo score) (**Table 1**; **Figures 1C, D**). H&E staining of mucosal tissues revealed inflammatory cell infiltration and loss of crypt architecture in UC, whereas the degree of inflammatory cell infiltration was markedly reduced in mild UC (**Figure 1B**).

**Table 1:** The clinical characteristics of samples.

Group		Control (*n* = 4)	Mild UC (*n* = 4)	Moderate–Severe UC(*n* = 4)	*P*
Age		49.5 ± 5.393	61.25 ± 5.662	50.5 ± 5.207	ns
Gender	Male	3	3	2	ns
	Female	1	1	2	
Disease duration (year)		/	6.25 ± 0.75	5.25 ± 1.377	ns
Extent of disease		/	E3	E3	/
Medical treatment			IFX	IFX	/
Mayo score		/	3.5 ± 0.479	9.75 ± 0.947	0.029
MES		/	0.5 ± 0.29	2.75 ± 0.25	0.029
PLT (10^9^/L)		/	165.3 ± 28.75	338.5 ± 50.66	0.025
WBC (10^9^/L)		/	4.503 ± 0.145	7.755 ± 1.166	0.029
ESR(mm/h)		/	21 ± 3.028	52.25 ± 9.835	0.023
Hb (g/L)		/	121± 6.819	129 ± 11.56	ns

IFX, infliximab; MES, Mayo Endoscopic Subscore; PLT, platelets; WBC, white blood cell count; ESR, Erythrocyte Sedimentation Rate; Hb, hemoglobin. The median represents the non-parametric test data, and the Means ± SEM represents the parametric test results. ns, *P* ≥ 0.05.

### 3.2 Integrated multi-omics data to identify UC-associated differentially ubiquitinated genes

**Figure 2:**
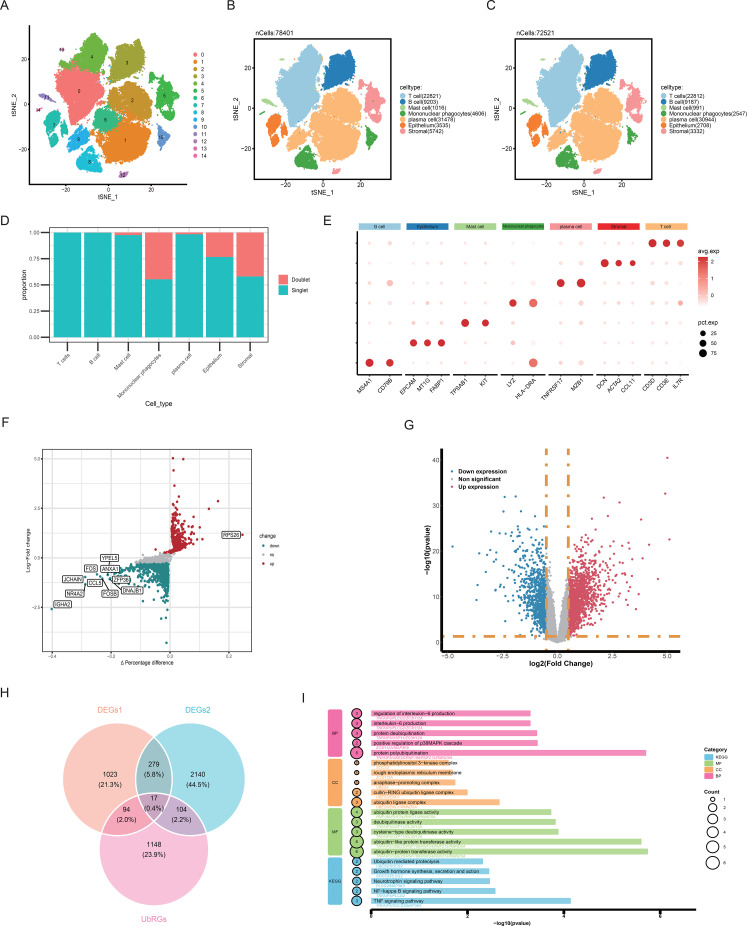
Identification of ubiquitination-related genes in UC. **(A)** t-SNE dimensionality reduction clustering of the integrated single-cell datasets (GSE214695, GSE182270). **(B–D)** Single-cell distributions and proportions of major cell populations before and after doublet removal. **(E)** Expression bubble plots of marker genes for the seven major cell subpopulations. **(F)** Volcano plot of DEGs1 between UC and control **(G)** Volcano plot of DEGs2 in the transcriptomic dataset (GSE87466). **(H)** Venn diagram of DEGs1, DEGs2, and UbRGs, identifying 17 core candidate genes. **(I)** Bar plots of GO and KEGG functional enrichment analyses for the 17 core candidate genes.

This study integrates single-cell datasets (GSE214695, GSE182270) and transcriptomic datasets (GSE87466), identifying key ubiquitination genes associated with UC. Construction of the single-cell atlas: the integrated single-cell data were subjected to t-SNE and identified 15 cell clusters (**Figure 2A**). After Doublet Finder identified and removed 7.5% of high-confidence doublets, 72,521 high-quality single cells were retained (**Figures 2B–D**). Referencing the Cell Marker database, cells were precisely annotated into seven major categories, including T cells (*CD3D^+^*, *CD3E^+^*, IL*7*R*^+^*), B cells (*CD79B^+^*, *MS4AI^+^*), epithelial cells (*EPCAM^+^*, *MT1G^+^*, *FABP1^+^*), mast cells (*TPSAB1^+^*, *KIT^+^*), mononuclear phagocytes (*LYZ^+^*, HLA-*DRA^+^*), plasma cells (*TNFRSF17^+^*, *MZB1^+^*), and stromal (*DCN^+^*, *ACTA2^+^*, *CCL11^+^*). Differential analysis showed 1,413 differentially expressed genes 1 (DEGs1) between UC patients and controls (**Figure 2F**). Using the limma package, the differential genes 2 (DEGs2) in the transcriptomic dataset amounted to 2,540 (**Figure 2G**). Intersecting DEGs1, DEGs2, and the ubiquitination-related gene set, 17 core candidate genes were finally identified (**Figure 2H**). The core candidate genes are significantly enriched in key inflammatory signaling pathways such as the TNF signaling pathway and NF-κB signaling pathway and ubiquitin-mediated proteolysis (**Figure 2I**), suggesting potential regulatory roles in the immunopathology of UC.

### 3.3 Screening of UC biomarkers

**Figure 3:**
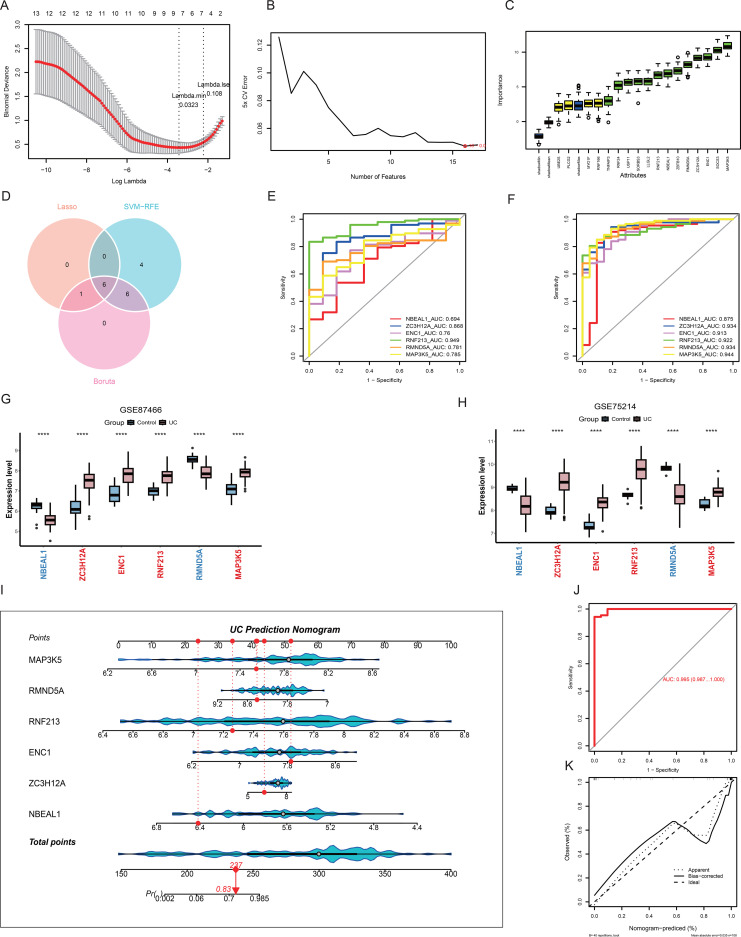
Machine learning-based selection of key biomarkers and construction of a diagnostic model. **(A)** LASSO regression analysis identifying feature genes. **(B)** SVM-RFE algorithm calculating cross-validation error rates across different feature counts. **(C)** Distribution of important variables selected by Boruta algorithm. **(D)** Venn diagram of results from the three machine learning methods, extracting six common genes. **(E, F)** ROC curves and diagnostic performance (AUC) for each biomarker in the training and validation cohorts. **(G, H)** Box plots comparing expression levels of the key biomarkers between controls and UC in the training set and the validation set. **(I)** UC Nomogram diagnostic model was constructed based on five key biomarkers. **(J)** ROC curve for the Nomogram diagnostic model. **(K)** Calibration curve for the Nomogram model, assessing the agreement between predicted probabilities and actual incidence.

Machine learning-based biomarker screening: LASSO regression selected seven feature genes (**Figure 3A**). SVM-RFE with the lowest error rate of 0.0472 selected 16 feature genes (**Figure 3B**). Boruta identified 13 important variables (**Figure 3C**). The intersection of the three methods yielded six common genes: *NBEAL1*, *ZC3H12A*, *ENC1*, *RNF213*, *RMND5A*, and *MAP3K5* (**Figure 3D**). ROC curves showed that six genes (*NBEAL1*, *ZC3H12A*, *ENC1*, *RNF213*, *RMND5A*, and *MAP3K5*) demonstrated excellent diagnostic performance in both the training and validation cohorts (AUC > 0.7), especially the AUC of RNF213, which was above 0.9 in both training and validation (**Figures 3E, F**). Coupled with significant differential expression analyses (**Figures 3G, H**), the final key biomarkers were identified as *ZC3H12A*, *ENC1*, *RNF213*, *MAP3K5*, and *RMND5A*. Based on these five genes, a nomogram diagnostic model was constructed (**Figure 3I**). Calibration curves indicated that predicted probabilities closely matched observed values, and the overall AUC exceeded 0.7, validating the model’s potential for clinical screening of UC (**Figures 3J, K**).

### 3.4 Localized the biomarkers in T-cell subpopulations

**Figure 4:**
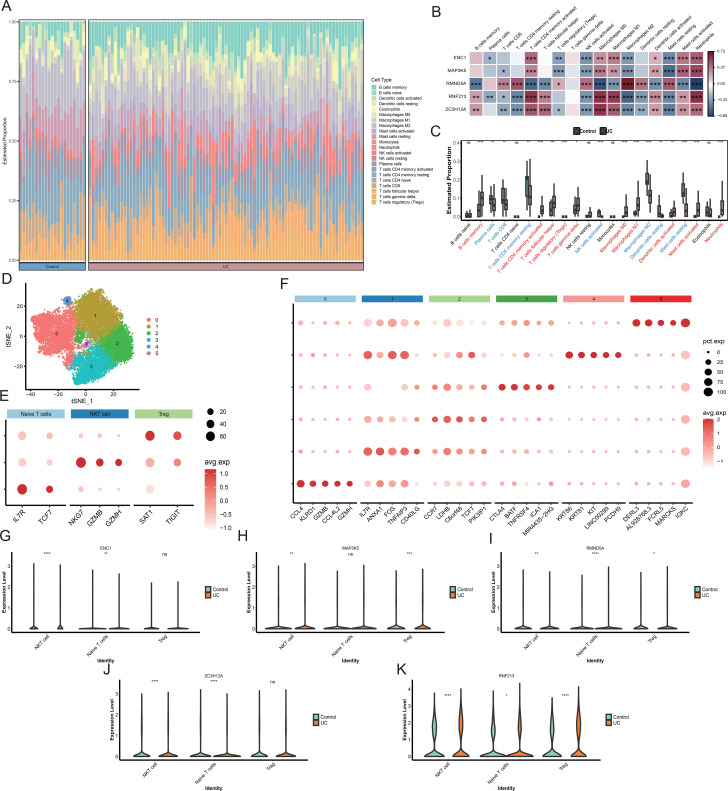
Localization of biomarkers in T-cell subpopulations. **(A)** Stacked bar chart of the proportions of immune cells from control and UC tissues assessed by CIBERSORTx. **(B)** Heatmap showing the correlations between the five key biomarkers and the infiltration levels of different immune cell subpopulations. **(C)** Box plots showing proportions of immune cell infiltration between control and UC groups. **(D)** t-SNE distribution after secondary clustering of T cell subpopulations. **(E, F)** Bubble plots illustrating the expression of characteristic marker genes for NKT cells, naive T cells (naive T), and regulatory T cells (Treg). **(G–K)** Violin plots of expression levels for the five key biomarkers (*ENC1*, *MAP3K5*, *RMND5A*, *ZC3H12A*, *RNF213*) across T-cell subpopulations. ^*^P < 0.05, ^**^P < 0.01, ^***^P < 0.001.

CIBERSORTx analysis shows significant dysregulation of 17 immune cell types in UC tissues (**Figure 4A**). Correlation analysis indicates that the biomarkers are significantly related to immune infiltration, with RNF213 showing a strong positive correlation with memory CD4 T-cell activation, macrophages M0, and neutrophils (**Figure 4B**). Furthermore, compared to the healthy control, UC exhibited increased proportions of memory B cells, activated memory *CD4*^+^ T cells, M0 macrophages, M1 macrophages, antigen-presenting cells, activated macrophages, and neutrophils, while decreased proportions were observed in *CD8*^+^ T cells and M2 macrophages (**Figure 4C**). T-cell subclustering and localization: recognizing the central role of T cells in mucosal immunity, NK T (NKT) cells, naive T cells, and Tregs were identified (**Figures 4D–F**). Expression profiling (**Figures 4G–K**) reveals that the five biomarkers are significantly upregulated across T-cell subpopulations in UC, with RNF213 showing the most pronounced expression in the Treg cell subset.

### 3.5 Validation of the findings by mIF

**Figure 5:**
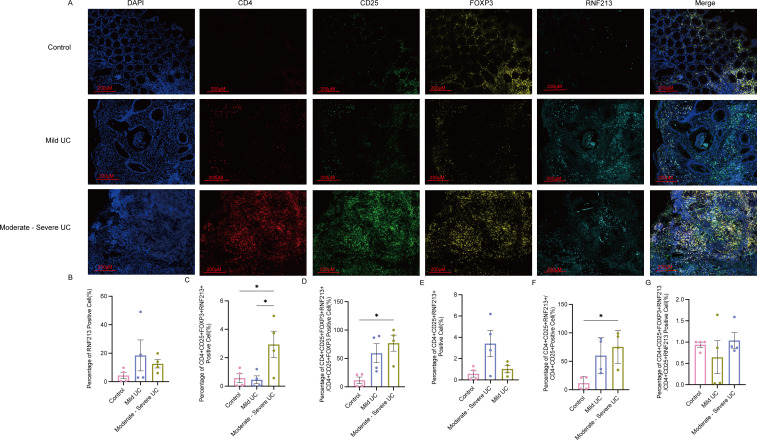
*In-situ* validation of RNF213 expression in Treg cells from UC patients by mIF. **(A)** Representative 20× single-channel and merged mIF images of human intestinal mucosa from normal control and UC. The images show DAPI (blue, nuclei), CD4 (red), CD25 (green), FOXP3 (yellow), RNF213 (cyan), and merged channels. **(B)** Quantification of RNF213^+^ cells. **(C)** Quantification of *RNF213*^+^ Tregs. **(D)** Quantification of RNF213 among total *CD4*^+^ T cells. **(E)** Quantification of *RNF213*^+^ Tregs among total Tregs. **(F)** Quantification of *RNF213*^+^ *CD4*^+^ T cells among total *CD4*^+^ T cells. **(G)** Quantification of *RNF213*^+^ Tregs among total *RNF213*^+^ *CD4*^+^ T cells.

mIF results demonstrated that in normal colon tissue (control), the fluorescence signals of *CD4^+^CD25^+^FOXP3^+^* regulatory T cells (Tregs) and RNF213 protein were both weak. However, in tissues from moderate-to-severe ulcerative colitis (moderate–severe UC), extensive immune cell infiltration was observed, with significantly enhanced fluorescence intensity for CD4, CD25, FOXP3, and RNF213, accompanied by distinct co-localization in merged images (**Figure 5A**). Quantitative statistical analysis further confirmed that the proportion of *CD4^+^CD25^+^FOXP3^+^RNF213^+^* positive cells among total cells in the moderate-to-severe UC group was significantly higher than in the control group and the mild colitis group (*P* < 0.05) (**Figure 5C**). The positive expression rate of RNF213 (*CD4^+^CD25^+^FOXP3^+^RNF213^+^*/*CD4^+^CD25^+^ FOXP3^+^*positive cell) in Tregs from moderate-to-severe UC was also significantly elevated compared to the control group (*P* < 0.05) (**Figure 5D**). Similarly, the proportion of *RNF213^+^CD4^+^* T cells (*CD4^+^CD25^+^RNF213^+^*/*CD4^+^CD25^+^*positive cells) among *CD4^+^* T cells also exhibited this trend (*P* < 0.05) (**Figure 5F**). These findings indicate that RNF213 expression is markedly activated in Tregs of the intestinal mucosa in UC patients, suggesting that RNF213 may play a role in UC.

### 3.6 Altered cell–cell communication in the UC microenvironment mediated by *RNF213^+^* Treg interactions

**Figure 6:**
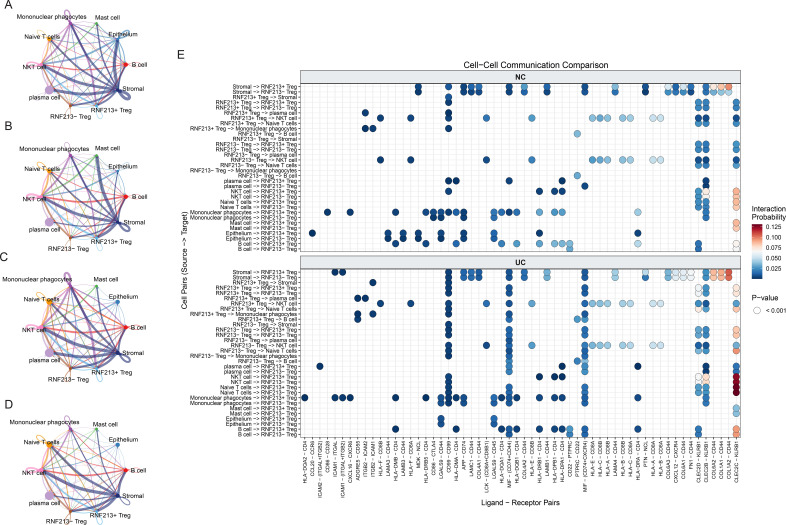
Predicted cell–cell communication patterns involving *RNF213^+^* Tregs in UC. **(A, B)** Network topology diagrams showing the number and strength of cell–cell interactions among mononuclear phagocytes, mast cells, naive T cells, NKT cells, B cells, and plasma cells, from healthy control (NC) individuals. **(C, D)** Network topology diagrams for UC, illustrating the global changes in communication strength. **(E)** Bubble plots comparing key ligand–receptor pairs mediating interactions between *RNF213*^+^ Treg cells and other cell populations. The bubble size reflects statistical significance (*P*-value), and the color intensity reflects the interaction probability.

The circular network diagram illustrates the global cell–cell communication network among diverse cell populations, including mononuclear phagocytes, mast cells, naive T cells, NKT cells, B cells, plasma cells, *RNF213*^+^ Treg cells, *RNF213*^-^ Treg cells, stromal cells, and epithelial cells (**Figures 6A–D**). Compared with the normal control (NC), the UC (**Figures 6A, C**) shows a more complex and denser connectivity, indicating enhanced intercellular interactions. The intermediate states represent transitional or subpopulation-specific communication patterns during UC progression, highlighting dynamic changes in cell–cell interactions (**Figures 6B, D**). Notably, *RNF213*^+^ Treg cells were central hubs in the UC network, forming broad connections with multiple cell types (e.g., mononuclear phagocytes, epithelial cells, stromal cells), whereas their interactions are relatively sparse in the NC group (**Figure 6E**), underscoring the central role of *RNF213*^+^ Treg cells in UC pathophysiology. In the NC group, *RNF213*^+^ Treg–involved interactions show moderate probabilities and relatively few statistically significant ligand–receptor pairs. In contrast, UC exhibits significantly increased communication involving *RNF213*^+^ Treg, with higher interaction probabilities and significance. For example, the MIF pathway (ligand: MIF; receptor: CD74/CXCR4) mediates stronger interactions between *RNF213^+^* Treg cells and mononuclear phagocytes in UC (higher probability, *P* < 0.001), whereas this pathway is not prominent in NC. Additionally, in UC, *RNF213^+^* Treg cells interact more strongly with epithelial cells (via LAMB3-ITGA1/ITGB1, LAMA5-CD44) and with stromal cells (via COL1A2-CD44, CLEC2B-KLRB1), suggesting involvement in tissue remodeling and inflammatory amplification. Other intercellular interactions also change significantly in UC: interactions between mononuclear phagocytes and B cells, NKT cells, and naive T cells intensify; communications between epithelial cells and stromal cells and plasma cells strengthen, potentially reflecting barrier dysfunction and immune activation.

CellChat analysis suggested that *RNF213^+^* Tregs may be involved in enhanced ligand-receptor interaction networks in UC. In particular, the MIF-CD74/CXCR4 axis was predicted to mediate communication between *RNF213^+^* Tregs and mononuclear phagocytes. These findings indicate a potential link between *RNF213^+^* Tregs and inflammatory immune crosstalk, although further validation using co-culture assays or ligand-receptor blockade experiments is warranted.

### 3.7 *In-silico* RNF213 perturbation suggests metabolic transcriptional changes in Tregs

**Figure 7:**
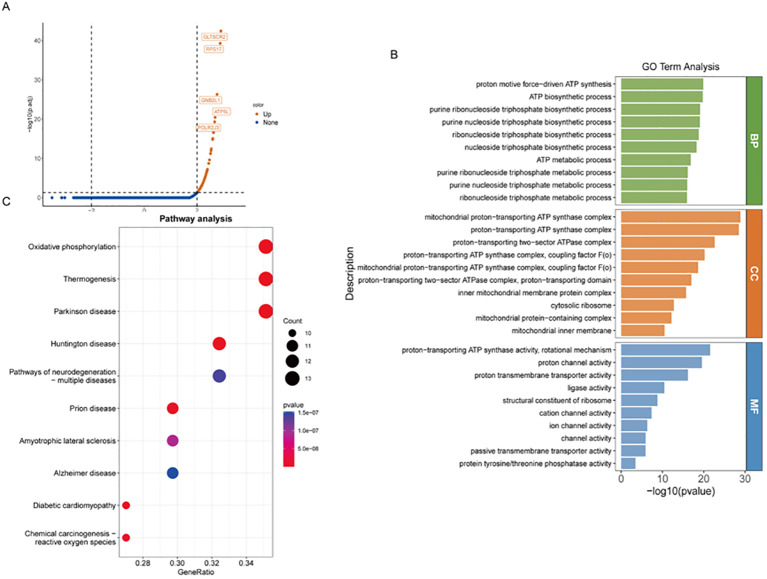
Virtual knockout–based exploration of RNF213 intracellular metabolic regulation in Treg cells. **(A)** Scatter plot of significantly differentially regulated genes after *RNF213* virtual knockout in Treg cells. **(B)** Bar plot of GO functional enrichment (including BP, CC, and MF). **(C)** Bubble plot of KEGG pathway enrichment for the differential genes.

To explore the intracellular function of RNF213, scTenifoldKnk virtual knockouts were performed on Treg cells. After RNF213 knockout, 88 genes were significantly upregulated (**Figure 7A**). Functional enrichment revealed that the affected genes are highly enriched in metabolic pathways such as ATP synthesis and mitochondrial proton transport (**Figure 7B**). KEGG pathway enrichment bubble plot of the differential genes, revealing potential impacts on key energy metabolism pathways such as oxidative phosphorylation (**Figure 7C**). These *in-silico* results suggest a potential association between *RNF213* and metabolic transcriptional programs in Tregs, particularly pathways related to oxidative phosphorylation and ATP synthesis. However, direct metabolic and functional validation remains necessary.

### 3.8 Predicted the potential targeted drugs and natural compounds for UC

**Figure 8:**
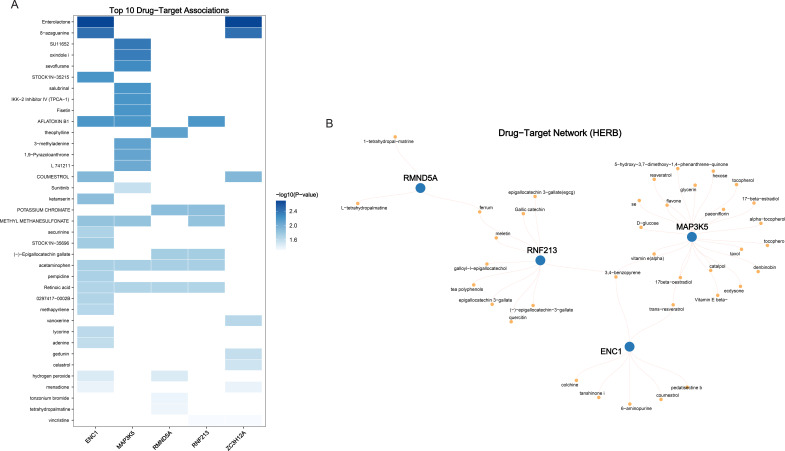
Predicting potential targeted chemical drugs and natural compounds for key biomarkers. **(A)** Top 10 potential chemical drugs associated with targeting the five key biomarkers predicted by the DSigDB database, shown as a heatmap (color intensity represents statistical significance, log_10_ *P*-value). **(B)** Drug–target interaction network of natural active constituents targeting the key biomarkers derived from the Herb database, illustrating multi-target regulation between monomer compounds and target genes.

Exploring the clinical translational potential of key biomarkers by integrating DSigDB and Herb databases. By screening five core genes for potential targeted drugs and natural active constituents using DSigDB in combination with the Herb database. In the small-molecule chemical drug domain (**Figure 8A**), a total of 72 potential intervention compounds were enriched. Among these compounds, MAP3K5 exhibited particularly high druggability and was identified as the intervention target for 49 compounds, followed by ENC1, targeted by 28 compounds. In natural product discovery (**Figure 8B**), 37 natural plant monomers or active constituents with potential therapeutic value were identified, among which RNF213 was targeted. These database-derived predictions generated a preliminary list of candidate compounds for prioritization in future experimental validation. Nevertheless, the present study did not include direct target-binding assays, pharmacodynamic evaluations, or efficacy validation experiments.

## 4 Discussion

Immune dysregulation plays a crucial role in the onset and progression of UC. An imbalance in T-cell homeostasis is closely associated with the development of inflammatory bowel disease (IBD) (13). Treg dysfunction leads to impaired self-tolerance and heightened inflammatory responses in IBD (14). T-cell homeostasis is maintained by multiple ubiquitin-modifying enzymes (UMEs) (15).

Studies have shown that deficiencies of E3 ubiquitin ligases USP8, SHARPIN, or VHL impair the generation and function of Treg cells (16). By integrating multi-omics results, we identified potential biological targets related to T-cell ubiquitination, including ZC3H12A, ENC1, RNF213, MAP3K5, and RMND5A, among which RNF213, an E3 ubiquitin ligase, offers the best diagnostic value for UC. Functional enrichment analysis of ubiquitination-related intersecting genes in T cells revealed significant enrichment in inflammatory signaling pathways such as TNF signaling, NF-κB signaling, IL-6 production, and regulation of IL-6 production. NF-κB, a dimeric transcription factor family, lies at the core of the IBD immune response and is a major driver of intestinal inflammatory abnormalities (17). Inflammatory cytokines TNF and IL-6 are closely related to UC onset and progression (18). Therefore, our results confirm that ubiquitination is not only involved in protein degradation but is also closely related to UC inflammatory signaling pathways and the maintenance of intestinal homeostasis.

RNF213 has previously been reported as a susceptibility gene for Moyamoya disease (19, 20), but its role in IBD has not been reported. Our study found that *RNF213*^+^ Treg cells in the colonic mucosa of UC show expression levels that correlate with disease activity, providing a new perspective for understanding UC pathogenesis.

When RNF213 function is lost, mitochondrial energy metabolism-related genes such as GLTSCR2, RPS17, GNB2L1, and ATP5L are significantly upregulated. GLTSCR2 is a pro-apoptotic protein related to energy metabolism (21). RPS17 is associated with autoimmune diseases such as systemic lupus erythematosus and rheumatoid arthritis, but there have been no reports in UC (22). GNB2L1, as a scaffolding protein, has been reported to promote CD74^+^ dendritic cell expansion when deficient, thereby promoting the progression of diabetic foot ulcers (23). ATP5L is a mitochondrial ATP synthase, and its function is closely linked to mitochondrial homeostasis (24). We hypothesize that RNF213 may regulate Treg cell energy metabolism in UC; functional enrichment analysis shows that mitochondrial proton transport and ATP synthesis pathways are significantly enriched in Treg cells, supporting this hypothesis. Mitochondrial dysfunction exacerbates UC-related intestinal barrier dysfunction and inflammation (25). Our previous work also confirmed that key energy metabolism enzymes HMGCS2 and AMACR are associated with mitochondrial dysfunction and UC progression (26). Previous studies reported that the E3 ubiquitin ligase RNF180 exacerbates colitis in mice by regulating the ALKBH5/SMARCA5 axis, leading to an imbalance in the intestinal milieu and Th17/Treg cells (27).

Interestingly, we pharmacologically studied the screened targets and identified 72 chemical drugs and 37 natural plant monomers. RNF213-targeting tea polyphenols such as epigallocatechin-3-gallate (EGCG), gallic catechin, galloyl-1-epigallocatechin, and other natural catechins exhibit strong anti-inflammatory and antioxidant capacities (28). We hypothesize that these natural products may modulate the function of RNF213 to alleviate UC, and in the future we will further pursue this research.

This study has certain limitations. Firstly, the specific molecular substrates and ubiquitination sites by RNF213 that regulate Treg cell mitochondrial metabolism require confirmation in future work via mass spectrometry, co-immunoprecipitation, and mutational analyses. Secondly, although virtual knockout predictions provide valuable mechanistic clues, *in-vivo* validation using RNF213 conditional knockout transgenic mice is needed to verify its biological function. Finally, the pharmacodynamics of natural compounds will require further evaluation in future cellular and animal experiments.

In summary, this study identifies RNF213 as a ubiquitination-related candidate biomarker enriched in UC-associated mucosal Tregs. Integrated transcriptomic, single-cell, mIF, in silico perturbation, and CellChat analyses suggest a potential association between *RNF213^+^* Tregs, metabolic transcriptional programs, and altered immune communication within the UC microenvironment. Nevertheless, the present findings are largely correlative and hypothesis-generating. Future studies involving RNF213 genetic manipulation, Treg suppressive assays, mitochondrial functional assays, ubiquitination-proteomic profiling, and validation in larger clinical cohorts are warranted to elucidate the biological role of RNF213 in UC.

## Data Availability

The original contributions presented in the study are included in the article. Further inquiries can be directed to the corresponding authors.
